# Potential therapeutic implications of IL-6/IL-6R/gp130-targeting agents in breast cancer

**DOI:** 10.18632/oncotarget.7102

**Published:** 2016-01-31

**Authors:** Tae-Hwe Heo, Joseph Wahler, Nanjoo Suh

**Affiliations:** ^1^ NP512, Laboratory of Pharmacoimmunology, Integrated Research Institute of Pharmaceutical Sciences, College of Pharmacy, The Catholic University of Korea, Seoul, Republic of Korea; ^2^ Department of Chemical Biology, Ernest Mario School of Pharmacy, Rutgers, The State University of New Jersey, Piscataway, NJ, USA; ^3^ Rutgers Cancer Institute of New Jersey, New Brunswick, NJ, USA

**Keywords:** breast cancer, interleukin-6, gp130

## Abstract

Interleukin-6 (IL-6) is a pleiotropic cytokine with known multiple functions in immune regulation, inflammation, and oncogenesis. Binding of IL-6 to the IL-6 receptor (IL-6R) induces homodimerization and recruitment of glycoprotein 130 (gp130), which leads to activation of downstream signaling. Emerging evidence suggests that high levels of IL-6 are correlated with poor prognosis in breast cancer patients. IL-6 appears to play a critical role in the growth and metastasis of breast cancer cells, renewal of breast cancer stem cells (BCSCs), and drug resistance of BCSCs, making anti–IL-6/IL-6R/gp130 therapies promising options for the treatment and prevention of breast cancers. However, preclinical and clinical studies of the applications of anti–IL-6/IL-6R/gp130 therapy in breast cancers are limited. In this review, we summarize the structures, preclinical and clinical studies, mechanisms of action of chemical and biological blockers that directly bind to IL-6, IL-6R, or gp130, and the potential clinical applications of these pharmacological agents as breast cancer therapies.

## INTRODUCTION

**Figure 1:**
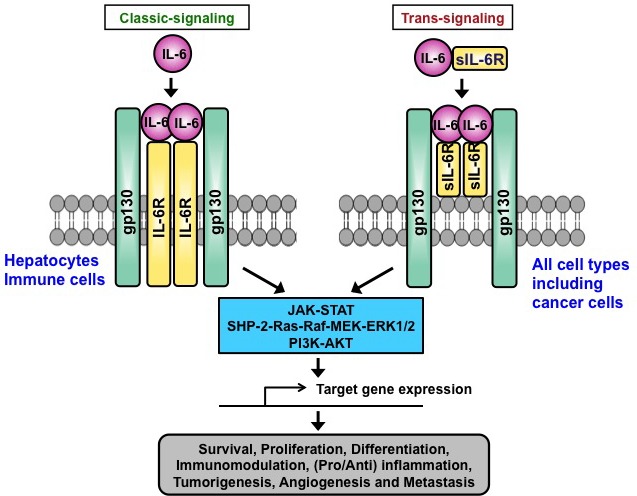
Action of IL-6 on target cells via classic-signaling and trans-signaling In classic signaling, binding of IL-6 to membrane IL-6R recruits gp130 to form an IL-6/IL-6R/gp130 hexamer complex. In trans-signaling, proteolysis and/or alternative splicing generates soluble form of IL-6R (sIL-6R), which binds to IL-6 and can stimulate gp130-expressing cells. Both classic and trans-signaling pathways activate downstream signaling including JAK-STAT, SHP-2-Ras-Raf-MEK-ERK1/2, and PI3K-AKT, leading to the transcription of target genes important for cell survival, proliferation, differentiation, immunomodulation, inflammation, angiogenesis, metastasis, and tumorigenesis.

Recent studies have demonstrated that the proinflammatory cytokine interleukin-6 (IL-6) plays an important role in tumor progression and metastasis [1-15]. IL-6 is a 23- to 30-kDa pleiotropic cytokine that is produced by various types of cells, including immune cells, fibroblasts, and certain tumor cells [13, 16, 17]. Upon binding of IL-6 to IL-6 receptor (IL-6R), the IL-6/IL-6R complex recruits glycoprotein 130 (gp130) to form a hexameric IL-6/IL-6R/gp130 complex composed of two IL-6, two IL-6R, and two gp130 subunits that initiate downstream signaling [13, 16-18] (Figure 1). This IL-6 signaling via the membrane IL-6R and gp130 has been termed classic-signaling. An alternative to classic-signaling has recently been described, termed trans-signaling, in which a complex is formed between IL-6 and a soluble form of IL-6R, sIL-6R, which then joining with membrane gp130 to generate downstream signaling events [19] (Figure 1). Classic-signaling is limited to a few cell types since membrane IL-6R is only expressed on hepatocytes and immune cells. In contrast, sIL-6R, which is generated by alternative splicing and/or proteolysis, can bind to IL-6 and elicit trans-signaling in all cells due to ubiquitous expression of membrane gp130 [20-22]. IL-6 plays an important role in immune responses and repair processes through classic-signaling, and may be involved in the pathogenesis of inflammatory diseases and cancers through trans-signaling. However, the full range of biological functions of IL-6 mediated by classic and trans-signaling remains to be elucidated [23].

Emerging evidence shows that IL-6 plays critical roles in cancer development, progression and metastasis by regulating the tumor microenvironment and cancer stem cells [7]. Because of this, the IL-6/IL-6R/GP130 signaling pathway represents an attractive target for therapeutic or preventive intervention. The application of IL-6 blockers as anticancer agents has been investigated in many cancer types, including lung cancer, multiple myeloma, B-cell non-Hodgkin's lymphoma, prostate cancer, renal cell carcinoma, ovarian cancer, and also in oral mucositis in head and neck cancer, and cachexia related to lung cancer [13]. Presently, however, only two monoclonal antibodies (mAbs), tocilizumab (anti–IL-6R) and siltuximab (anti–IL-6), have been approved in the US for the treatment of rheumatoid arthritis (RA) and Castleman's disease respectively, but not for cancer. In this review, we evaluate the evidence regarding potential use of IL-6 antagonists for breast cancer therapy and prevention.

## THE ROLE OF IL-6 IN BREAST CANCER DEVELOPMENT

IL-6 is expressed in approximately 50% of breast cancers [24]. Increasing evidence supports the notion that high levels of serum IL-6 are associated with poor prognosis, advanced disease, and metastases in breast cancer patients [6, 13, 25-28]. Breast cancer is commonly classified by receptor expression and can be categorized into estrogen receptor positive (ER+), HER2-positive, or triple-negative breast cancer [29]. The three breast cancer subtypes rely on IL-6 signaling to varying degrees.

Approximately 75% of breast cancer cases are ER+ [30]. ER+ breast cancer can be successfully treated with selective estrogen receptor modulators, such as tamoxifen, but drug resistance eventually occurs in most patients [31]. ER+ breast cancer patients tend to have lower than average serum levels of sIL-6R when compared to ER- breast cancer patients [32]. Patients with above average serum levels of sIL-6R are more likely to experience recurrence compared to patients with lower levels of sIL-6R [32]. The transcription factor HOXB13 enhances progression and recurrence of breast cancer by down-regulation of ERα and up-regulation of IL-6 expression [31]. IL-6 treatment of the estrogen receptor-expressing cell line, MCF-7, showed a Notch-3 dependent up-regulation of the Notch ligand, Jagged-1, and the carbonic anhydrase IX [33]. IL-6 also promoted a hypoxia-resistant and invasive phenotype in MCF-7 cells [33]. Secretion of IL-6 by cancer-associated fibroblasts has also been implicated in the suppression of ERα and tamoxifen resistance in luminal breast cancer cell lines [34]. Therefore, combination therapy with ER antagonists and IL-6/IL-6R/gp130 inhibitors may provide a novel therapeutic strategy for ER+ breast cancers.

The HER2 gene is amplified in approximately 25-30% of breast cancer cases [35]. IL-6 is elevated in HER2-positive breast cancer where IL-6 activated STAT3 and induced an autocrine loop of IL-6/STAT3 expression [36]. Further evidence revealed that the growth of HER2-positive breast cancer *in vivo* was dependent on the HER2/IL-6/STAT3 signaling pathway [36]. Drug resistance is a critical problem in breast cancer therapy, and autocrine production of IL-6 by breast tumor cells promotes resistance to multi-drug chemotherapy [37]. Very recently, IL-6 has been suggested as a major factor influencing resistance to trastuzumab, a therapeutic HER2 antibody, in breast cancer [38]. Trastuzumab resistance in HER2-overexpressing breast cancer cells is shown to be mediated by the IL-6 inflammatory loop, leading to expansion of the breast cancer stem cell population [38]. Blockade of this IL-6 loop by an IL-6 antagonist, tocilizumab, reduced the cancer stem cell population, resulting in decreased tumor growth and metastasis in mouse xenografts [38]. Further studies are warranted to assess the potential of utilization of HER2 therapies in combination with IL-6 therapies to overcome drug resistance in HER2-positive breast cancers.

Triple-negative breast cancer, one of the most aggressive forms of the disease, accounts for approximately 10-20% of breast cancer cases [29, 39]. In comparison to other breast subtypes, triple-negative breast cancer cell lines secret the highest levels of IL-6 [40]. Triple negative breast cancers rely on the autocrine expression of IL-6 for growth [40]. Studies have shown that inhibition of IL-6 expression by shRNA in triple-negative breast cancer cells can lead to the suppression of colony formation and decreased cell survival *in vitro* as well as decreased tumor engraftment and growth *in vivo* [40]. Induction of IL-6 production by the adipokine leptin in breast cancer amplifies STAT3 signaling, and phosphorylation of STAT3 is significantly reduced by IL-6 neutralizing antibodies [41]. With limited therapy options for aggressive triple-negative breast cancer, IL-6 signaling inhibitors may offer an important new therapeutic option.

IL-6 signaling not only exerts its effects on breast cancer cells, but can also play a role in the surrounding tumor microenvironment, indirectly impacting cancer growth and progression [42]. The tumor microenvironment is composed of various cell types including mesenchymal stem cells, adipocytes, tumor-associated fibroblasts, endothelial cells, and immune cells, all of which are capable of interaction with tumor cells via cytokine networks [43]. Both autocrine and paracrine actions of IL-6 in the tumor microenvironment are reported to be critical for breast oncogenesis [6, 43]. IL-6 produced by tissue-specific fibroblasts promotes the growth and invasion of breast cancer cells through STAT3-dependent up-regulation of Notch-3, Jagged-1, and carbonic anhydrase IX [44, 45]. STAT3 phosphorylation in breast epithelial cells can be stimulated by paracrine signaling through IL-6 from both breast cancer cells and fibroblasts [46]. IL-6 secreted from senescent mesenchymal stem cells can increase the proliferation and migration of breast cancer cells by induction of STAT3 phosphorylation [14]. Utilizing IL-6 signaling inhibitors to target the tumor microenvironment and indirectly block cancer cell growth could be effective in treating and preventing breast carcinogenesis.

## DIRECT IL-6 BINDING ANTAGONISTS

**Figure 2:**
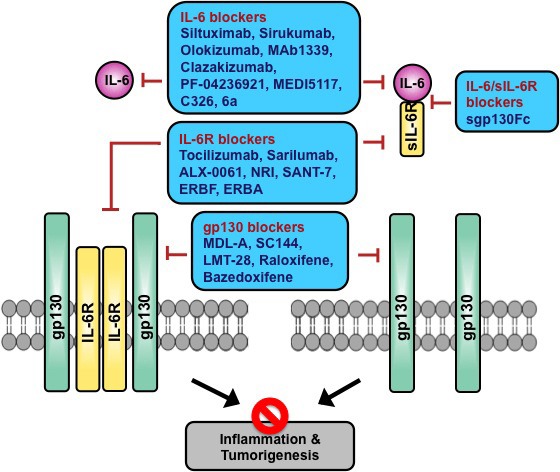
Potential targets for inhibiting IL-6-induced inflammation and tumorigenesis by IL-6/IL-6R/gp130 blockers IL-6 inhibitors, such as anti-IL-6 mAbs and anti-IL-6 avimers, block the binding of IL-6 to both membrane IL-6R and extracellular sIL-6R. IL-6R inhibitors, including anti-IL-6R mAb, anti-IL-6R scFv, and anti-IL-6R nanobody can bind to IL-6R and inhibit both classic and trans-signaling. Soluble form of gp130 Fc fusion protein (sgp130Fc) is a decoy antagonist of IL-6 trans-signaling via binding to the IL-6/sIL-6R complex. Anti-gp130 mAb and anti-gp130 chemical compounds bind to gp130 and inhibit IL-6-induced gp130 dimerization and signaling.

There are four potential extracellular targets to antagonize IL-6 signaling, IL-6 itself, IL-6R, gp130, and/or IL-6/sIL-6R complex. Recently developed IL-6 targeting agents include chimeric, humanized or human monoclonal antibodies (mAbs), avimers, and small molecules (Figure 2). Currently available IL-6/IL-6R/gp130 blockers are summarized in Table 1, and are discussed in detail in this section.

**Table 1:** Agents directly targeting the IL-6/IL-6R/gp130 complex for cancer therapy

Name; Developer	Structure	Preclinical study	Clinical study	Proposed mechanisms of action
IL-6 targeting agents				
Siltuximab (CNTO 328, Sylvant); Centocor/J&J	Chimeric anti–IL-6 mAb	- Inhibition of IL-6–induced ovarian cancer [49] - Inhibition of IL-6–induced prostate cancer [57] - Enhancement of melphalan cytotoxicity in multiple myeloma [51]	- Multiple myeloma (phase II) [56, 99] - Prostate cancer (phase II) [48, 100] - B-cell Non-Hodgkin Lymphoma (phase I) [52] - Renal cell cancer (phase I/II) [54] - Ovarian cancer (phase II) [5]	- Inhibition of IL-6 signaling and enhancement of paclitaxel sensitivity [49] - Increased apoptosis sensitivity via induction of myeloid cell leukemia 1 (Mcl-1) [57] - Enhanced activation of caspase with melphalan [51]
Sirukumab (CNTO 136); J&J/GSK	Human anti–IL-6 mAb	N/A	- Pharmacokinetic, pharmacodynamics, and safety study in healthy subjects (phase I) [61]	- Binding and neutralization of human IL-6 with high affinity and specificity [13]
Olokizumab (CDP6038); UCB	Humanized anti–IL-6 mAb	N/A	- Pharmacokinetic and safety study in healthy subjects (phase I) [64]	- Binding and neutralization of human IL-6 with high affinity and specificity [13]
MAb 1339 (OP-R003); OPi EUSA/Vaccinex/GSK	Human anti–IL-6 mAb	- Inhibition of growth of multiple myeloma in vitro and in vivo [66]	N/A	- Inhibition of IL-6 signaling, such as phosphorylation of STAT3, ERK1/2, and Akt [66]
Clazakizumab (BMS945429, ALD518); Alder/BMS	Humanized anti–IL-6 mAb	- Ablation of acute phase reaction in rats induced by IL-6 [68]	- Safety study was carried out in patients with various cancers (phase I) [68] - Randomized, blinded, placebo-controlled study involving 124 non-small cell lung cancer patients (phase II) [68]	- Strong binding affinity for human IL-6 (KD=4 pM) [68]
PF-04236921; Pfizer	Humanized anti–IL-6 mAb	N/A	N/A	- Binding to human IL-6
MEDI 5117; AstraZeneca	Human anti–IL-6 mAb	N/A	N/A	- Binding to human IL-6
C326 (AMG-220); Avidia/Amgen	Anti–IL-6 avimer protein	N/A	N/A	- Specificity and avidity against human IL-6
6a	Anti–IL-6 synthetic pyrrolidinesulphonylaryl compound	- Inhibition of phosphorylation of STAT3 in IL-6 stimulated MDA-MB-231 breast cancer [70]	N/A	- Selective inhibition of STAT3 phosphorylation [70]
IL-6R targeting agents				
Tocilizumab (Actemra, RoActemra); Roche/Chugai	Humanized anti–IL-6R mAb	- Amelioration of mouse model of lung cancer cachexia by a rodent analog of tocilizumab (MR16-1) [101] - Inhibition of fibrosarcoma growth in vivo by MR16-1 (74) [75] - Anti-colon cancer effect in xenograft model [9] - Anti-renal carcinoma effect of co-administration with interferon [77]	- Case reports of amelioration of cancer cachexia by the administration of tocilizumab [74, 102]	- Milder cachectic parameters by targeting IL-6 [101] - Restoration of CD4+ T cell function by inhibition of IL-6 activity leading to downregulation of arginase-1 and upregulation of MHC class II on dendritic cells [75] - Anti-angiogenic activity by affecting stromal cells (6) - Suppression of SOCS3 expression [77]
Sarilumab (REGN88,SAR153191); Regeneron/Sanofi-Aventis	Human anti–IL-6R mAb	- Inhibition of the growth of xenograft tumors with active IL6/STAT3 signaling, both as a single agent and in combination with aflibercept [78]	N/A	- Binding to human IL-6R and preventing binding of IL-6, thereby inhibiting downstream signaling [78]
ALX-0061; Ablynx/AbbVie	Anti–IL-6R nanobody	N/A	- Bioavailability, pharmacokinetics, pharmacodynamics, safety, and immunogenicity in healthy volunteers (phase I) (trial ID: NCT02101073*)	- A bi-specific nanobody that targets IL-6R and serum albumin [23]
NRI	Anti–IL-6R single chain Fv of tocilizumab fused to IgG1 Fc	- Reduction of multiple myeloma cells (S6B45) in vivo by injection of adenovirus vector encoding NRI [80]	N/A	- Sustained therapeutic concentration of NRI in circulation and comparable to parental tocilizumab in terms of inhibitory activity [80]
SANT-7	Super-antagonist of IL-6; rationally designed mutant of IL-6	- Anti-multiple myeloma effects in vitro and in vivo [82-84] - Potentiation of the action of dexamethasone against multiple myeloma in a SCID-hu in vivo model [85], and enhancement of effects of dexamethasone and all-trans-retinoic acid in multiple myeloma cell line [84]	N/A	- Inhibition of wild type IL-6 by preoccupancy of IL-6R [81] - Cell cycle arrest and induction of apoptosis [84, 85]
ERBF (20S,21-epoxy-resibufogenin-3-formate)	A natural compound with anti–IL-6R-antagonist activity	- Suppression of IL-6-induced growth of tumor cell line [86]	N/A	- Suppression of IL-6 binding to IL-6R [86]
ERBA (20S,21-epoxy-resibufogenin-3-acetate)	A semi-synthetic derivative of ERBF with anti–IL-6R-antagonist activity	- Suppression of IL-6 activities and alleviation of cancer cachexia [87]	N/A	- Suppression of IL-6 binding to IL-6R [87]
gp130 targeting agents				
B-R3	Anti-gp130 mAb	N/A	N/A	- Inhibitory effects on IL-6-induced gp130 homodimerization and the downstream signaling [89]
B-P4	Anti–gp130 mAb	- Inhibition of the constitutive activation of naturally occurring gp130 mutants in inflammatory hepatocellular adenoma [90]	N/A	- Blockade of gp130-induced STAT3 phosphorylation in hepatic adenomas [90]
MDL-A (Madindoline A)	Anti-gp130 natural compound	- Inhibition of non-small-cell lung cancer cell xenografts in nude mice with combination of MDL-A and crizotinib [56]	N/A	- Inhibition of the IL-6/STAT3 and PI3K/AKT/mTOR pathways [56]
SC144 (quinoxalinhydrazide derivative)	Anti-gp130 synthetic compound	- Inhibition of tumor growth of human ovarian cancer xenografts [94]	N/A	- Suppression of STAT3 signaling via induction of gp130 phosphorylation and downregulation of gp130 glycosylation [94]
Raloxifene	Anti-gp130 synthetic compound	- Inhibition of IL-6-induced breast cancer cell (SUM159) proliferation [97]	- Breast cancer prevention (phase III/II) (NCT00003906*) (NCT00190593*) (NCT00019500*)	- Inhibition of constitutive STAT3 phosphorylation in human breast cancer cell line possibly by disrupting IL-6/gp130 interface [97] - Complete estrogen antagonist in mammary gland (clinical study)
Bazedoxifene	Anti-gp130 synthetic compound	- Inhibition of IL-6-induced breast cancer cell (SUM159) proliferation [97]	- Changes in breast density (NCT00774267*)(NCT00418236*)	- Inhibition of constitutive STAT3 phosphorylation in human breast cancer cell line possibly by disrupting IL-6/gp130 interface [97] - Complete estrogen antagonist in mammary gland (clinical study)
LMT-28 ((4R)-3-((2S,3S)-3-hydroxy-2-methyl-4-methylenenonanoyl)-4-isopropyldihydrofuran-2(3H)-one)	Anti-gp130 synthetic compound	- Inhibition of IL-6-induced STAT3 activation and cell proliferation [88] - Anti-inflammatory activity in vivo [88]	N/A	- Direct binding to gp130 [88] - Inhibition of IL-6 signaling, including phosphorylation of JAK2 and STAT3 and binding of IL-6 as well as soluble IL-6/IL-6R complex [88]
IL-6/sIL-6R complex targeting agents				
sgp130Fc (FE 999301); Conaris/Ferring	Soluble gp130 linked to IgG-Fc	- Suppression of colon carcinogenesis in TGF transgenic mice [103] - Significant reduction of the colitis-associated premalignant cancer in mice [72]	N/A	- Inhibiting signaling via the sIL-6R, but not the membrane bound IL-6R [103] - Suppressing the activation of STAT3, NFκB, and gp130 via the IL-6/sIL-6R complex [72]

* Information from http://www.clinicaltrials.gov. Abbreviations: N/A, not applicable. References are provided in parenthesis.

### Siltuximab (CNTO 328, Sylvant); Centocor/Johnson & Johnson (J&J)

Siltuximab (CNTO 328) is a chimeric anti–IL-6 mAb that has recently been approved for multicentric Castleman's disease, a rare B-cell lymphoproliferative disorder [47]. Siltuximab has been shown to be potentially effective in the treatment of various cancers as a single agent or in combination with other anti-cancer drugs [48-56]. In preclinical studies, siltuximab inhibited IL-6–induced activation of ovarian cancer cells [49], inhibited IL-6–induced survival of advanced prostate cancer [57], suppressed lung cancer growth in mouse xenograft models [55], and enhanced melphalan cytotoxicity in a preclinical multiple myeloma model [51]. A case report showed that complete remission was achieved in a patient with relapsed refractory multiple myeloma after single agent therapy with siltuximab [58]. Clinical trials of siltuximab for development as a drug for smoldering multiple myeloma are ongoing; however, its development for advanced multiple myeloma, hormone-refractory prostate cancer, ovarian cancer, non-Hodgkin's lymphoma, and renal cancer has been discontinued due to lack of efficacy in clinical trials [53]. The addition of siltuximab to bortezomib did not result in improved outcome in patients with relapsed or refractory multiple myeloma [59]. Lack of anti-multiple myeloma effect of siltuximab in clinical trials could be explained by emergence of IL-6-independent subclones or substitution for IL-6 by other IL-6 family cytokines that utilize gp130 as a shared signal transducer [60]. To date, no preclinical or clinical data are available for breast cancer therapy; however, the safety and efficacy profile of siltuximab is well established, making it ideal for future studies.

### Sirukumab (CNTO 136); Centocor/J&J/GlaxoSmithKline (GSK)

Sirukumab, a human anti–IL-6 mAb, has been found to bind to human IL-6 with high affinity and specificity, and to suppress IL-6 activity. Pharmacokinetics (PK), pharmacodynamics (PD), and safety profile studies of sirukumab in healthy subjects have revealed good tolerability, desirable PK characteristics, and low immunogenicity [61]. Sirukumab has been studied in patients with rheumatoid arthritis (RA), systemic lupus erythematosus (SLE), and cutaneous lupus erythematosus [13]. Clinical efficacy of sirukumab was evaluated in a phase II study of patients with active RA failing methotrexate monotherapy and showed improvements in the symptoms of RA [62]. Due to desirable PK/PD characteristics, sirukumab would be a prime candidate for testing in breast cancer. Sirukumab is a human mAb, and therefore it has an advantage of a very low immunogenicity in anti-cancer therapies.

### Olokizumab (CP6038); UCB

Olokizumab, a humanized anti–IL-6 mAb, has been studied in patients with RA [13]. Crystal structure data have revealed that binding of olokizumab to site 3 (the gp130 binding site) on IL-6 induces a conformational change in IL-6 and neutralizes its biological activity by blocking receptor hexamer complex formation [63]. A phase I pharmacokinetic and safety study in healthy subjects was performed and showed no serious adverse events and rapid decreases in C-reactive protein concentration [64]. On the basis of the pathogenetic role of IL-6 in cancers, a study of the anti-cancer activity of olokizumab appears warranted.

### mAb 1339 (OP-R003); OPi EUSA/Vaccinex/GSK

mAb 1339 is a fully human version of murine mAb B-E8 (Elsilimomab) directed against human IL-6 [65]. mAb 1339 inhibited the growth of multiple myeloma *in vitro*, and synergistically enhanced the cytotoxic activity of dexamethasone *in vivo* in a SCID-hu mouse model of multiple myeloma [66]. Given its inhibitory role against multiple myeloma, mAb 1339 may have potential activity against other types of cancer, including breast cancer.

### Clazakizumab (BMS945429, ALD518); Bristol-Myers Squibb/Alder Biopharmaceuticals

The safety and efficacy of the humanized anti–IL-6 mAb clazakizumab was evaluated in a phase II clinical trial in patients with active RA [67] and a phase I/II clinical trial in patients with glucocorticoid-refractory acute graft versus host disease (NCT01530256). For cancer therapy, clazakizumab was evaluated for the treatment of non-small cell lung cancer (NSCLC)-related fatigue and cachexia and oral mucositis in patients with head and neck cancer [13, 68]. In preclinical and clinical trials, clazakizumab ameliorated NSCLC-related anemia and cachexia [68]. No preclinical or clinical data are currently available for the activity of clazakizumab against breast cancer.

### PF-04236921; Pfizer

PF-04236921, a humanized anti-IL-6 mAb, is under clinical trial for Crohn's disease, RA, and SLE [13]. A clinical study to assess the efficacy and safety of PF-04236921 in subjects with Crohn's disease who failed anti-TNF therapy is ongoing (NCT01287897). PF-04236921 has not been used clinically or preclinically for anti-cancer therapy.

### MEDI 5117; AstraZeneca

MEDI 5117, a human anti–IL-6 mAb, is under clinical trial for RA [13]. A clinical study (NCT01559103) to assess the safety and tolerability of MEDI 5117 in RA patients was terminated due to difficulties with patient recruitment. Thus far, no patients were reported to have severe adverse events. No other activity of MEDI 5117 except for RA has been suggested.

### C326 (AMG-220, Anti-IL-6/anti-IgG avimer protein); Amgen/Avidia

Avimers are created from a large family of human extracellular receptor domains by exon shuffling and phage display to generate multidomain proteins with binding properties that may overcome the limitations of mAbs [69]. C326 is composed of an IL-6–binding trimer and a IgG-binding domain, resulting in a heterotetrameric avimer with very high affinity for IL-6 and in vivo neutralizing activity. A phase 1 clinical study of the safety and biological effects of C326 in Crohn's disease is ongoing (NCT00353756).

### 6a; University of London, England

6a is a pyrrolidinesulphonylaryl synthetic molecule that suppresses IL-6 signaling in the MDA-MB-231 breast cancer cell line via selective inhibition of STAT3 phosphorylation and transcription [70]. Docking studies identified potential binding sites for 6a at the protein–protein interfaces of IL-6/gp130 or IL-6/IL-6R complexes [70]. Further studies are underway to identify the precise site of action of 6a. Molecular mechanism studies in breast cancer cell lines warrant further investigation of 6a as an anti-cancer therapeutic.

### sgp130Fc (Soluble gp130-Fc fusion protein, FE 999301); Ferring/conaris

Soluble gp130 (sgp130) is a natural antagonist selective for IL-6 trans-signaling, which is found in serum and binds to IL-6/sIL-6R complex without affinity for IL-6 or IL-6R alone [22]. A current IL-6/sIL-6R targeted molecule under study is soluble gp130 linked to IgG-Fc (sgp130Fc) (Figure 2). Soluble gp130Fc (sgp130Fc) showed competitive inhibition of IL-6/sIL-6R–induced trans-signaling [71]. Treatment with sgp130Fc significantly reduced colitis-associated premalignant cancer in mice [72]. Because of its specific blockade of proinflammatory trans-signaling [23], sgp130Fc has potential as an effective and safe therapeutic strategy for breast cancer.

## DIRECT IL-6R BINDING ANTAGONISTS

Structures of current IL-6R targeting molecules include humanized or human mAbs, bi-specific nanobody, single chain Fv (scFv) linked to IgG-fragment crystallizable (Fc), IL-6 mutants, and small molecules (Figure 2). Generally, it has been shown that clinical efficacy as well as safety profiles among anti-IL-6 and anti-IL-6R mAbs appear similar in RA patients [73]. Currently available IL-6R blockers are discussed in this section and summarized in Table 1.

### Tocilizumab (Actemra, RoActemra); Roche/Chugai

Tocilizumab, a humanized anti–IL-6R mAb that blocks both soluble IL-6R and membrane-bound IL-6R, has been approved by the FDA for the treatment of RA and shown to be an effective therapy for other inflammatory diseases, including Castleman's disease [13]. MR16-1, a rodent analog of tocilizumab, exhibited a dramatic effect on cachexia induced by an IL-6–overexpressing lung cancer [74]. MR16-1 inhibited fibrosarcoma growth *in vivo* [75]. MR16-1 treatment enhanced antitumor activity through the elimination of myeloid-derived suppressor cells and enhancement of CD8^+^ and CD4^+^ cell responses [75, 76]. Recent reports showed anti-cancer effects of tocilizumab in a colon cancer xenograft model [9] and the effects of combination therapy of tocilizumab and interferon-alpha against renal cell carcinoma [77]. Tocilizumab also inhibited tumor growth of trastuzumab resistant breast cancer cells [38]. However, so far no clinical study has been reported in breast cancer.

### Sarilumab (REGN88, SAR153191); Regeneron/Sanofi-Aventis

Sarilumab, a human anti–IL-6R mAb, is under clinical trial for RA and ankylosing spondylitis [13]. The results of a phase II study to assess the safety and efficacy of sarilumab in active RA patients have been reported [73]. Sarilumab showed efficacy in patients with active RA compared to placebo [73]. For cancer therapy, inhibition of the growth of xenograft tumors of DU145 (prostate), Calu3 (lung), and A549 (lung) cells by sarilumab was reported both as a single agent and in combination with the VEGF blocker aflibercept [78]. The potential of sarilumab in cancer inhibition has been demonstrated, however, its effects in breast cancer are unknown and should be investigated.

### ALX-0061 (nanobody); Ablynx/AbbVie

ALX-0061 is a bi-specific nanobody that targets IL-6R and serum albumin [23]. Its small size (26 kD) should allow ALX-0061 to penetrate more effectively into tissues [79]. A phase I/II study of ALX-0061 was completed in February 2013, and demonstrated strong efficacy and safety in patients with moderate to severely active RA on a stable background dose of methotrexate (NCT01284569).

### NRI; Osaka University, Japan

NRI is an anti–IL-6R single chain Fv (scFv) of tocilizumab fused to IgG1 fragment crystallizable (Fc). Injection of an adenovirus vector encoding NRI exhibited inhibitory effects on multiple myeloma cells (S6B45) in vivo [80]. This study showed a sustained therapeutic concentration of NRI in the circulation and inhibitory activity comparable to that of the parent agent tocilizumab.

### SANT-7; Institute of Research in Molecular Biology (IRBM)

SANT-7 is a rationally designed mutant of IL-6 and a super-antagonist of IL-6 [81]. SANT-7 showed activity against multiple myeloma *in vitro* and *in vivo* [82, 83]. Combination therapy with SANT-7 and dexamethasone or all-*trans*-retinoic acid showed activity against multiple myeloma in a SCID-hu *in vivo* model and in cell lines [84, 85].

### ERBF; Kitasato University, Japan

ERBF (20*S*,21-epoxy-resibufogenin-3-formate) is a small molecule with IL-6R–antagonist activity that suppresses the binding of IL-6 to IL-6R. Although evidence of direct interaction between ERBF and IL-6/IL-6R has not been demonstrated, ERBF suppressed IL-6–induced neuronal differentiation and osteoclast formation [86]. The anti-differentiation potential of ERBF suggests that it could be utilized in anticancer therapies.

### ERBA; Kanagawa University, Japan

ERBA (20S,21-epoxy-resibufogenin-3-acetate) is another small molecule with IL-6R–antagonist activity that suppresses the binding of IL-6 to IL-6R. Although evidence of direct interaction between ERBA and IL-6/IL-6R is lacking, ERBA specifically suppressed IL-6 activities and alleviated cancer cachexia [87].

## DIRECT GP130 BINDING ANTAGONISTS

Although increasing attention has recently been paid to the role of gp130 in cancer, no pharmaceutical company-driven anti-gp130 agents are currently under development. Anti-gp130 mouse mAbs and some chemical small molecules have been reported (Figure 2). However, mouse mAbs are not eligible for human clinical studies and some small molecule inhibitors have not been studied in cancer. Most recently, direct binding of a novel small molecule inhibitor to gp130 has been demonstrated [88]. gp130 antagonists could be a new effective class of drugs which target IL-6 signaling to treat cancer.

### B-R3 and B-P4; CHRU Angers

B-R3 is a mouse mAb against gp130 that has been used in preclinical studies to inhibit gp130 signaling [22, 89]. Another anti-gp130 mAb, B-P4, blocked gp130-induced STAT3 phosphorylation in hepatic adenomas [90]. No clinical trials are underway with anti-gp130 mAbs yet.

### Madindoline A (MDL-A); Kitasato University, Japan

MDL-A is a non-peptide antagonist of gp130 that shows IL-6/IL-6R blocking properties [91]. MDL-A suppressed osteoclastogenesis induced by IL-6 or IL-11 *in vitro* [91]. MDL-A binds to the extracellular domain of gp130 [92]. MDL-A analogues were synthesized and evaluated for their inhibitory activity against IL-6–dependent cell proliferation [93]. Several MDL-A analogues were identified as candidates for future development of IL-6-targeting inhibitors [93]. To date, no preclinical or clinical data are available for its activity against breast cancer.

### SC144; University of Southern California

SC144 is a small-molecule gp130 inhibitor that suppresses STAT3 signaling via induction of gp130 phosphorylation and down-regulation of gp130 glycosylation [94]. SC144 inhibited tumor growth of human ovarian cancer xenografts and reduced the number of tumor blood vessels [94]. Evidence of direct interaction between SC144 and gp130 was provided by drug affinity responsive target stability (DARTS) assay [94], and to date, no preclinical or clinical data are available for breast cancer therapy.

### Raloxifene (Keoxifene, LY156758, Evista); Eli Lilly and bazedoxifene (Viviant); Wyeth Pharmaceuticals

The selective estrogen receptor modulators (SERMs), raloxifene and bazedoxifene, are used clinically to treat or prevent ER+ invasive breast cancer and osteoporosis [95, 96]. Recently, raloxifene and bazedoxifene were discovered to be inhibitors of the IL-6/gp130 interface [97]. The interaction of these drugs with gp130 was demonstrated indirectly via docking studies and a drug affinity responsive target stability assay. Both agents inhibited IL-6–induced STAT3 phosphorylation in the pancreatic cancer cell line PANC-1 [97]. To date, no clinical data are available for breast cancer therapy. However, these SERMs may have potential effects against breast cancer via IL-6 signaling in addition to ER-modulating mechanisms.

### LMT-28; The Catholic University of Korea and Korea University, South Korea

A novel synthetic compound, LMT-28 [(4R)-3-((2S,3S)-3-hydroxy-2-methyl-4-methylenenonanoyl)-4-isopropyldihydrofuran-2(3H)-one], has been shown to bind directly and specifically to gp130, and thereby inhibits the binding and signaling of IL-6 and soluble complex of IL-6/IL-6R [88]. Oral administration of LMT-28 ameliorated collagen-induced arthritis and acute pancreatitis in mice. This compound possesses a potential to be used as an anti-cancer agent, targeting gp130 and is now being studied for anti-breast cancer activity.

## PERSPECTIVES ON THE APPLICATION OF DIRECT IL-6/IL-6R/GP130 BLOCKERS IN BREAST CANCER

Numerous IL-6/IL-6R/gp130 blockers have been described and developed as anti-inflammatory drugs, and some of them have also been studied as anticancer agents. However, preclinical and clinical studies on the effectiveness of IL-6 targeting agents for breast cancer are limited despite the substantial body of evidence indicating that high levels of IL-6 contribute to the poor prognosis and promotion of breast cancer. Among the IL-6 targeting agents, raloxifene and bazedoxifene, have been studied as anti-breast cancer agents based on a mechanism involving antagonizing estrogen action. A novel mechanism of gp130 targeting action was reported for raloxifene and bazedoxifene [97], however further studies with these agents are necessary to determine whether their key action is through direct interaction with gp130.

Although IL-6 targeting therapies have shown promising outcomes in inflammatory diseases and some types of cancers, safety issues related to IL-6 targeting approaches have been reported [23]. Adverse events associated with inhibition of IL-6 signaling include susceptibility to infection, cardiovascular toxicity, and gastrointestinal perforation [23]. Understanding the basic biology of IL-6, as well as large-scale and long-term clinical trials of new blocking agents of IL-6 signaling, will be important to reduce these risks.

Anti–IL-6 mAbs have achieved great commercial success to date, however, mAb biologics have some limitations in their clinical utility [79]. First, the production process is labor intensive and costly because of the complex molecular structure of mAbs. Second, invasive routes of administration are required. Third, penetration and accumulation of mAbs in tissues, including solid tumors and bone marrow, and accessibility to some epitopes on certain proteins, can be problematic due to their large size. Fourth, the instability of mAbs necessitates storage under refrigeration. Finally, the immunogenicity of some mAbs can abolish their effectiveness [98]. Other protein scaffolds, such as avimers and nanobodies with improved affinity and accessibility respectively, have been developed to overcome some of the limitations of mAbs [79]. Nevertheless, small molecule-based anti-IL-6/IL-6R/gp130 agents offer distinct advantages and could provide more versatility and flexibility as therapeutics.

## CONCLUSIONS

Interventions against the IL-6/IL-6R/gp130 signaling axis appear to be quite effective for some inflammatory diseases. At present, the application of IL-6/IL-6R/gp130 blockers as anti-cancer agents has not been studied broadly, and to an even lesser extent for breast cancer. However, considering the clinical development of several IL-6-based anticancer drugs, their application in oncology is likely to expand in the near future. Because of the reliance of disease progression on a variety of IL-6–related mechanisms in breast cancer, the IL-6 signaling pathway is an ideal target for drug development. Additional studies into the effects of IL-6 blockers in breast cancer are thus warranted and could offer exciting new directions for treatment. The critical challenges will be to identify which candidates will most effectively repress IL-6 signaling in breast cancer without adverse effects. With an increased understanding of the IL-6/IL-6R/gp130 regulatory axis and signaling in breast cancer, it may be possible to design new IL-6 therapies which will be more effective.
